# *In vitro *synergistic cytoreductive effects of zoledronic acid and radiation on breast cancer cells

**DOI:** 10.1186/bcr1543

**Published:** 2006-08-22

**Authors:** A Ugur Ural, Ferit Avcu, Muhammed Candir, Metin Guden, M Ali Ozcan

**Affiliations:** 1 Department of Hematology, Gulhane Military Medical Faculty, Ankara, Turkey; 2 Department of Medical and Cancer Research Center, Gulhane Military Medical Faculty, Ankara, Turkey; 3 Department of Internal Medicine, Gulhane Military Medical Faculty, Ankara, Turkey; 4 Department of Radiation Oncology, Gulhane Military Medical Faculty, Ankara, Turkey; 5 Department of Hematology, Medical Faculty of Dokuz Eylul University, Izmir, Turkey

## Abstract

**Introduction:**

Bisphosphonates are mostly used in the treatment of bone metastases. They have been shown to act synergistically with other chemotherapeutic agents. It is not known, however, whether similar synergistic effects exist with radiation on breast cancer cells.

**Methods:**

Human MCF-7 breast cancer cells were treated with up to 100 μM zoledronic acid, were irradiated with up to 800 cGy or were exposed to combinations of both treatments to determine the antiproliferative effects of zoledronic acid and radiation.

**Results:**

Zoledronic acid and radiation caused a dose-dependent and time-dependent decrease in cell viability (approximate 50% growth inhibition values were 48 μM and 20 μM for 24 hours and 72 hours, respectively, for zoledronic acid and 500 cGy for radiation). A synergistic cytotoxic effect of the combination of zoledronic acid and radiation was confirmed by isobologram analysis.

**Conclusion:**

These data constitute the first *in vitro *evidence for synergistic effects between zoledronic acid and radiation. This combination therapy might thus be expected to be more effective than either treatment alone in patients with metastatic breast carcinoma.

## Introduction

Breast cancer metastases in bone account significantly for morbidity and mortality, and can cause intractable pain, spinal cord compression and hypercalcemia. The survival of women who have solely bone metastases as the predominant, or only, metastatic site may be measured in years, indicating the important therapeutic need for an effective bone-specific palliative treatment [1]. This also signifies that the malignant process is incurable since, once tumor cells become lodged in the skeleton, therapy can only be given with palliative intent. This includes analgesics, radiation therapy and systemic treatments such as bisphosphonates (BPs) or chemotherapy.

Metastases from most cancer types are predominantly osteolytic, except for those from prostate cancer, which at least radiographically appear as sclerotic secondary lesions. Osteolysis is primarily a result of increased activity in osteoclasts, resulting in elevated calcium levels in blood. The BPs are pyrophosphate analogs used to treat osteoclast-mediated bone diseases, including osteoporosis, Paget's disease, hypercalcemia of malignancy, bone metastases and bone disease associated with multiple myeloma [2]. Current views suggest that BPs may affect the differentiation and recruitment of osteoclast precursors or may alter the capability of mature osteoclasts to resorb bone by altering the permeability of the osteoclast membranes to small ions [3]. The high concentrations of BPs that accumulate in bone are taken up by osteoclasts, specifically inhibit ATP-dependent enzyme systems, induce apoptosis of osteoclasts by inhibiting the mevalonic acid pathway, and thus disrupt the biochemical process that leads to bone destruction.

We recently demonstrated that zoledronic acid, one of the most potent nitrogen-containing BPs, induces antiproliferative and apoptotic effects on multiple myeloma cell lines *in vitro*, by activating protein kinase C, and increases the extracellular calcium concentration, these effects being augmented with dexamethasone and thalidomide [4]. Although several years ago the molecular target of the potent nitrogen-containing BPs was identified as farnesyl diphosphate synthase, an enzyme in the mevalonate pathway, recent data have shed new light on the precise mechanism of inhibition and have demonstrated that the acute-phase reaction, an adverse effect of BPs, is also caused by inhibition of this enzyme [5].

In another study, we demonstrated that zoledronic acid was able to increase disease-free survival in pristane-induced plasmacytoma, a model with no direct bone involvement, in BALB/c mice [6]. In that study, zoledronic acid treatment markedly impeded intraperitoneal plasmacytoma development. The treatment also decreased the tumor burden and extramedullary tumor growth in mice. Other studies have also suggested that BPs can inhibit the adhesion of breast cancer cells to bone matrices and can enhance the ability of antineoplastic agents to inhibit breast cancer cell invasion [7,8].

The demonstration that these BPs can induce apoptosis in cells other than osteoclasts, and that the treatment of cancer patients with BPs may improve survival, therefore raises the intriguing possibility that BPs may also have direct antitumor effects in breast cancer cells. Zoledronic acid is approved in the United States and in Europe for the treatment of bone metastases associated with breast cancer, prostate cancer, lung cancer and renal cancer [9]. Zoledronic acid was also found to be superior to pamidronate for the treatment of bone metastases in breast carcinoma patients with at least one osteolytic lesion [10].

Another well-established treatment modality for the local treatment of bone metastases is radiotherapy. Although the treatment effects of radiation for this application are primarily the result of the direct destruction of tumor cells, it has recently been shown that the effectiveness of radiotherapy could partially be explained by the inhibition of osteoclastic activity [11]. BPs have been shown to act synergistically with other chemotherapeutic agents in breast cancer cells [12], which further supports the notion that combined use of radiotherapy and BP might be even more effective than either treatment alone. To investigate this possibility we have now examined the cytotoxic effects of zoledronic acid and radiation on MCF-7 breast cancer cells, both alone and in combination.

## Materials and methods

The local ethics committee of Gulhane School of Medicine approved the study.

### Cell line and culture conditions

The human MCF-7 breast cancer cell line was generously supplied by the Molecular Pharmacology and Therapeutics Department, Memorial Sloan-Kettering Cancer Center, New York, USA. MCF-7 cells were maintained in RPMI 1640 medium supplemented with 10% heat-inactivated fetal calf serum, and with 2 mM L-glutamine, 100 μg/ml streptomycin and 100 U/ml penicillin in a 37°C incubator with a humidified air and 5% CO_2 _atmosphere. The culture medium was changed twice a week. Cells were removed from the flasks by treatment with trypsin–ethylenediamine tetraacetic acid.

### Reagents

Zoledronic acid was kindly supplied as the hydrated disodium salt by Novartis, Pharma AG (Basel, Switzerland). Zoledronic acid was dissolved in <0.1% dimethyl sulfoxide (DMSO) and diluted in culture medium (0.1–100 μM) immediately before use. The DMSO concentration in the assay did not exceed 0.1%. For control of possible adverse effects of the DMSO vehicle, medium alone also was used as a negative control in all experiments. In all studies, DMSO and media-alone cultures were equivalent. All other reagents were purchased from Sigma (St Louis, MO, USA).

### Measurement of cell number and percentage viability

MCF-7 breast cancer cells were resuspended in medium at 1 × 10^5 ^cells/ml, and 100 μl of 10^5 ^cells/ml cell suspension were distributed into each well of 96-well plates (Costar, Cambridge, MA, USA) and allowed to adhere for 24 hours. Cells were thereafter incubated with increasing concentrations of zoledronic acid (0.1–100 μM) and were treated for 24 and 72 hours to determine a 50% growth inhibition (IC_50_) value for zoledronic acid. In additional experiments, MCF-7 cells were irradiated with doses of 200, 400, 600, and 800 cGy with a Theratron 780 Cobalt 60 Teletherapy Unit (AECL Medical, Ontario, Canada), with or without incubating the cells, with the stated concentrations of zoledronic acid for 24 and 72 hours to determine an IC_50 _value for radiation and to construct the isobologram analyses. After irradiation, all plates were incubated at 37°C in a 5% CO_2 _atmosphere for an additional 24 hours. No medium changes were performed.

In some studies, target cells were plated at a density of 5 × 10^4 ^per well in 24-well plates with replicates of four wells per treatment, and were allowed to adhere for 24 hours. Cells were then incubated with fresh media containing 20 μM zoledronic acid (IC_50 _value for 72 hours) for 96 hours to determine the time dependence of zoledronic acid toxicity. In each separate experiment, following incubation, cells were harvested and suspended in PBS, and the cell number was determined using a haemocytometer (Bright-line; Hausser Scientific, Horseam, PA, USA). Each experiment was repeated on three occasions.

### MTT assay

The 3-(4,5-dimethyl-thiazoyl)-2,5-diphenyl-SH-tetrazolium bromide (MTT) assay was performed as previously described [4]. Target cells were resuspended in medium at 1 × 10^5 ^cells/ml, and 100 μl of 10^5 ^cells/ml cell suspension were distributed into each well of 96-well plates (Costar) and allowed to adhere for 24 hours. Wells containing 200 μl medium alone without cells and reagents were used as the negative controls.

After treatment for the stated incubation doses of zoledronic acid and radiation, and for the stated times, 20 μl MTT solution (5 mg/ml) was added to each well, and the microplates were further incubated at 37°C for 4 hours. The unreactive supernatants in the well were discarded, and 100 μl acidified isopropanol (0.04 N HCl in isopropanol) was added to the cultures and mixed thoroughly to dissolve the dark-blue crystals of formazan. The absorbance values of each well were determined with a microplate enzyme-linked immunoassay reader equipped with a 570 nm filter. The negative control well was used for the baseline zero absorbance.

The results are presented as the percentage viability, determined as (1 - absorbance of the experimental well/absorbance of the positive control well) × 100. Each experiment was repeated on three occasions.

### Isobologram analysis

The isobologram method of analysis was used to assess whether synergistic or additive effects were seen when zoledronic acid and radiation were combined, as described previously [13]. Dose–response curves were plotted for the effects of zoledronic acid and for the effects of radiation on MCF-7 cell viability. From these curves, the combined drug IC_50 _and 75% growth inhibition (IC_75_) values (e.g. 75% of maximum effect, 25% remaining) were determined for each curve. The ratio of the combined drug IC_50 _and IC_75 _values to the separate IC_50 _and IC_75 _values for each drug alone were calculated and plotted as an isobologram.

The combination index (CI) was then calculated by the formula: CI = (*d*1/*D*_*x*_1) + (*d*2/*D*_*x*_2), where *D*_*x*_1 is the dose of agent 1 (radiation) required to produce *x *percentage effect alone, and *d*1 is the dose of agent 1 required to produce the same *x *percentage effect in combination with *d*2. *D*_*x*_2 is similarly the dose of agent 2 (zoledronic acid) required to produce *x *percentage effect alone, and *d*2 is the dose of agent 2 required to produce the same *x *percentage effect in combination with d1. The CI values were interpreted as follows: <1.0, synergism; 1.0, additive; and >1.0, antagonism. Each experiment was repeated on three occasions.

### Statistical analysis

Data are reported as the mean ± standard deviation. Statistical analysis was carried out using SPSS software (version 11; SPSS Inc., Chicago, Illinois, USA). The two-tailed Student *t *test was used to determine statistical significance of detected differences. *P *< 0.05 was considered statistically significant.

## Results

### Effects of zoledronic acid and radiation on breast cancer cell growth

**Figure 1:**
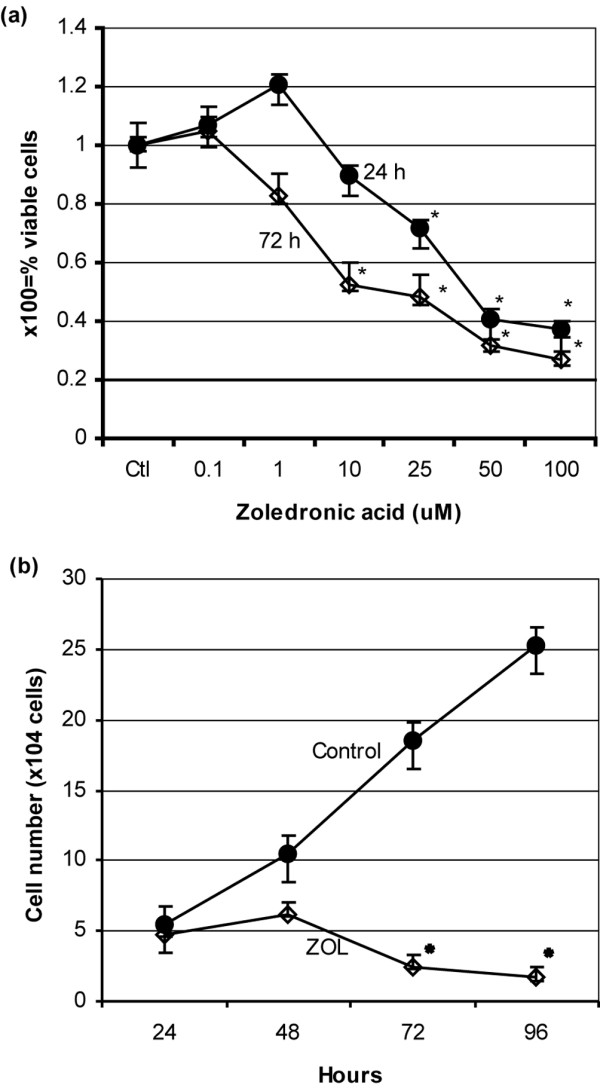
Effect of zoledronic acid treatment on MCF-7 cell viability. **(a) **MCF-7 cells treated with increasing concentrations of zoledronic acid (0.1–100 μM) for 24 and 72 hours to determine a 50% growth inhibition (IC_50_) value. (b) MCF-7 cells treated with 20 μM zoledronic acid for increasing time of incubation to determine the time dependence of zoledronic acid (ZOL) toxicity. Results are expressed as the percentage of control or absolute cell numbers. *Statistically significant values compared with control. Ctl, control.

Treatment of MCF-7 cells with increasing concentrations of zoledronic acid for 24 and 72 hours caused a dose-dependent and time-dependent decrease in cell viability (Figure 1a). Zoledronic acid (0.1–1 μM) had little effect on the viability of MCF-7 cells at 72 hours incubation. In contrast, zoledronic acid at concentrations higher than 10 μM caused a reduction in the viability of MCF-7 cells compared with controls (10% reduction at 10 μM and 63% reduction at 100 μM, *P *> 0.05 and *P *< 0.01, respectively, for 24 hours incubation; and 47.5% reduction at 10 μM and 73% reduction at 100 μM, *P *< 0.05 and *P *< 0.01, respectively, for 72 hours incubation).

**Figure 2:**
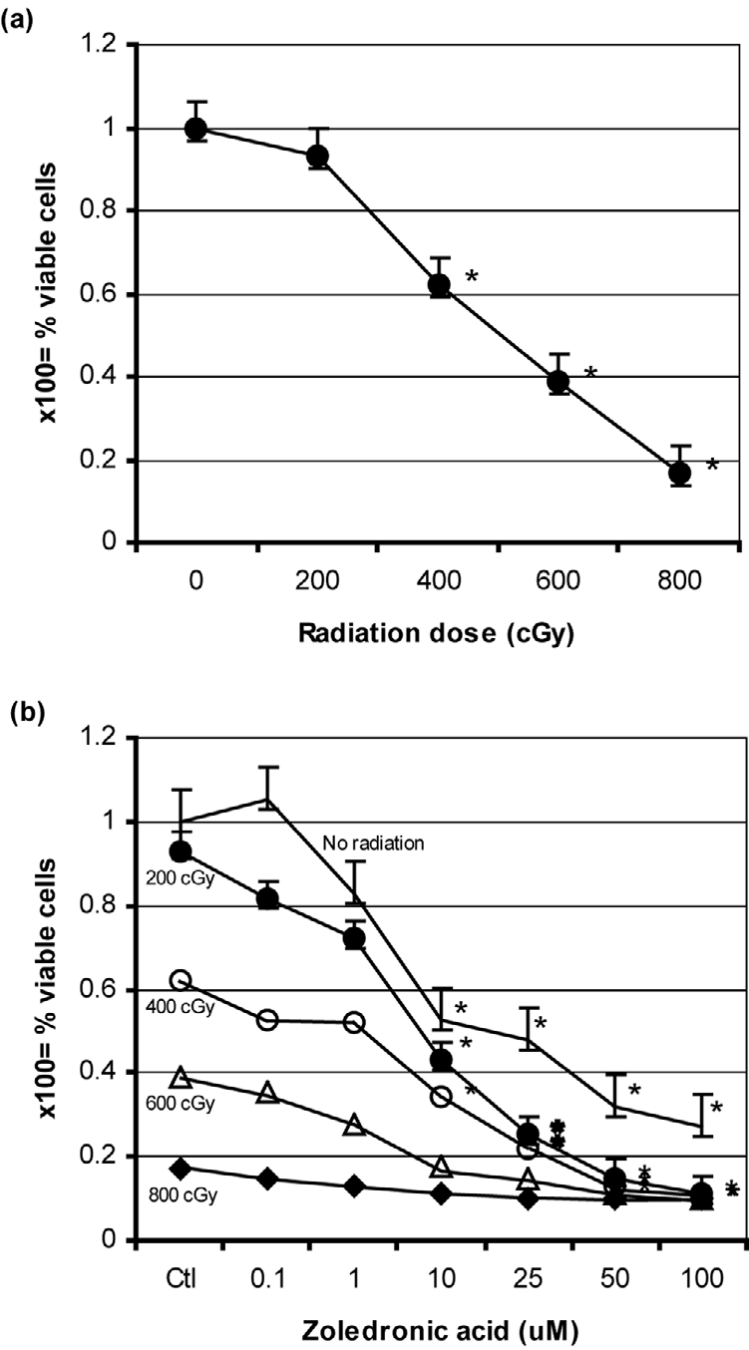
Radiation dose effect on MCF-7 cells. **(a) **MCF 7 cells treated with radiation alone. **(b) **The cytotoxic effect of radiation on MCF-7 cells increased steadily after incubating the cells with higher zoledronic acid concentrations. Ctl, control.

The approximate IC_50 _values for zoledronic acid were 48 μM and 20 μM for 24 hours and 72 hours, respectively. Treatment of breast cancer cells with 20 μM zoledronic acid for increasing periods of time resulted in a 86.5% reduction in MCF-7 cell growth at 72 hours and in a 93.3% reduction of control at 96 hours (*P *< 0.01 in each case) (Figure 1b). Radiation also caused a dose-dependent decrease in cell viability. The calculated IC_50 _value for radiation on MCF-7 cells was around 500 cGy. Figure 2a shows that higher radiation doses result in successively lower cancer cell survival.

### Combination of zoledronic acid and radiation

**Figure 3:**
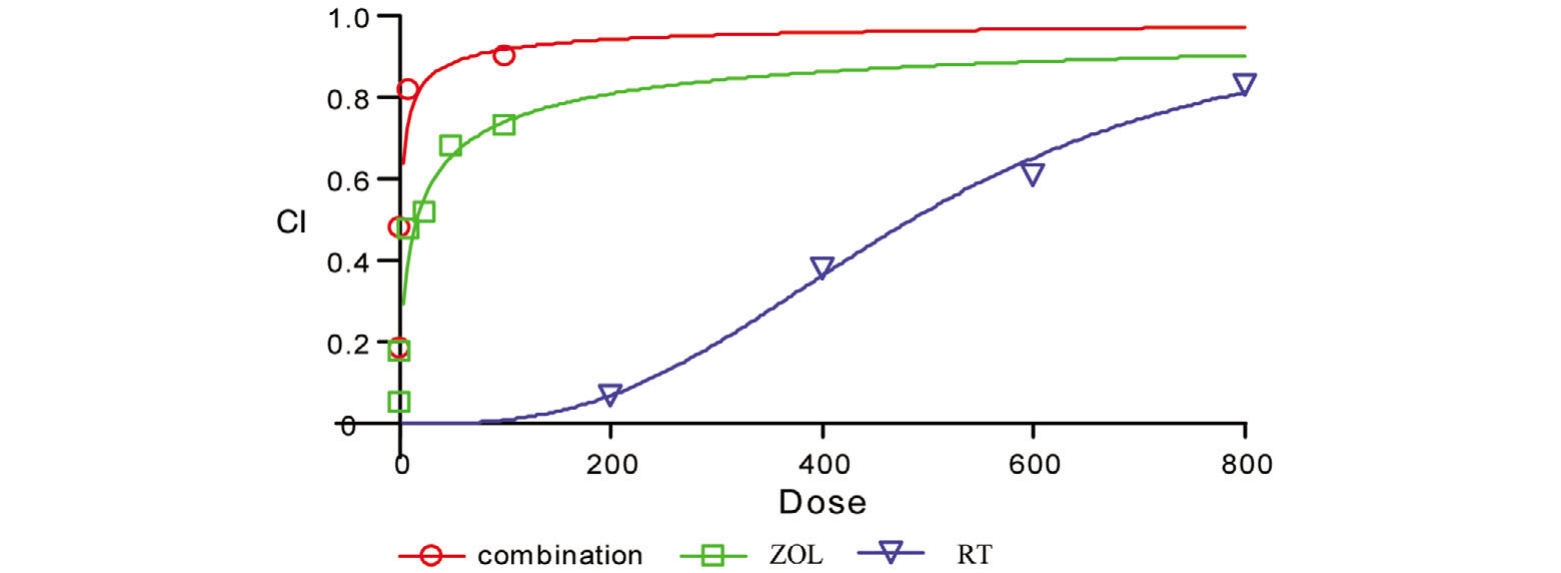
The combination index (CI) for the cell treatments with zoledronic acid (ZOL) and radiation (RT) for 24 and 72 hours. CI < 1.0 clearly points to a synergistic effect.

Breast cancer cell viability was inhibited by either zoledronic acid or radiation over the 1–100 μM and 200–800 cGy ranges, respectively. A combination of radiation with zoledronic acid caused a greater reduction in cell viability than did either treatment on its own (Figure 2b). Treatment of breast cancer cells with radiation alone induced a 75% growth inhibition (IC_75 _value) from approximately 600 cGy to 800 cGy doses. The IC_75 _concentration for zoledronic acid alone was in the range of 100 μM for 24 and 48 hours incubation. Compared with the single agent, the IC_75 _values for the dose pairs (radiation and zoledronic acid together for both time exposures) fall below 1.0, indicating that the combination is synergistic (Figure 3).

## Discussion

Our study demonstrates a dose-dependent and time-dependent cytotoxic effect of zoledronic acid on breast cancer cells *in vitro*. These results are consistent with earlier reports that zoledronic acid can inhibit breast cancer cell proliferation and invasion, and can induce apoptosis [14-16]. These studies reported cell growth inhibition levels closer to our results, between 50 and 100 μM.

Current trends in the treatment of human tumors are with drug combination. This approach results in improved responses as well as the ability to use lower, less toxic concentrations of the drugs. In clinical practice, metastatic breast cancer patients would rarely be treated with BPs alone, and may instead be given drug combinations such as anthracyclines and taxoids. The antitumor effect of BPs can therefore also be enhanced by coadministration of chemotherapeutic agents. We recently showed that zoledronic acid inhibits cell growth and synergistically induces apoptosis with dexamethasone and thalidomide on multiple myeloma cells [4]. In another study, combined treatment with alendronate and paclitaxel showed a greater effect on bone metastatic and nonbone metastatic development than treatment with each agent alone [17], while a synergistic effect on apoptosis was reported after coadministration of ibandronate or zoledronic acid with chemotherapeutic agents on breast cancer cells *in vitro *[12,16]. A most exciting recent observation is that sequential treatment of two drugs is even better than simultaneous treatment [18]. This notion, the synergistic antitumor effect of zoledronic acid with other chemotherapeutic agents, further supports the combined use of BPs and radiotherapy. We have shown that using the combination of zoledronic acid and radiation always synergistically enhanced growth inhibition on breast cancer cells compared with each agent alone.

The results for the combination of zoledronic acid and radiation are novel and intriguing because they immediately suggest the clinical utility of using zoledronic acid combined with radiation especially in patients with metastatic breast cancer. The combination proved to be true synergism rather than a simple additive effect, as shown clearly by the calculated CI (<1.0) (Figure 3). Algur and colleagues showed previously that increasing the incubation time with zoledronic acid to yield the optimal BP concentration during irradiation enhanced the synergistic effect of zoledronic acid and radiation on prostate and myeloma cell lines [19]. The synergistic effect might therefore be greater if the concentration and kinetics of the drugs are further optimized.

The biochemical mechanism of action of the BPs is now well documented, with nitrogen-containing BPs such as zoledronic acid affecting cellular behavior through inhibition of the mevalonate pathway [2]. In addition, the identification of BP analogs that inhibit different enzymes in the mevalonate pathway could lead to the development of novel inhibitors of bone resorption with potential applications in the treatment of metastatic bone disease [5].

The goal of radiotherapy is to eradicate malignant cells without damaging surrounding normal cells. Radiotherapy is usually delivered only to the immediate lesion area, to spare bone marrow and normal soft tissue as far as possible. In bone metastases, the endpoint of clinical success is pain palliation and enhancement of bone strength. The synergistic mechanism of zoledronic acid and radiation, however, is currently unknown. We recently showed by flow cytometric analysis of zoledronic acid-treated MM cells an increase in the proportion of cells in the S-phase, possibly due to a slower progression through the S-phase or a block between the S-phase and G_2_M-phase in the cell cycle [4]. Using small-cell lung cancer cell lines, Matsumoto and colleagues reported that cell growth inhibition may involve not only induction of apoptosis, but also prolongation of cell cycle progression by zoledronic acid alone or combined with anticancer agents [20]. This ability of zoledronic acid to arrest cells in the G_2_M phase or to prolong cell cycle progression raises the possibility of zoledronic acid as a potential cell cycle radiosensitizer because G_2 _and M cells are more sensitive than cells within other cell cycle phases [21].

Human cancers are often characterized by Ras mutations that lead to the constitutive activation of the Ras signaling pathway. Effective Ras signaling requires the attachment of Ras proteins to the plasma membrane, a process initiated by the enzyme farnesyl protein transferase. The blockade of Ras binding to the plasma membrane may therefore be a good therapeutic target for the treatment of malignancies [22]. Third-generation BPs deplete the cellular pool of both geranylgeranyl pyrophosphate and farnesyl pyrophosphate, and thereby inhibit both geranylgeranylation and farnesylation [23]. Salomo and colleagues reported that BP-resistant cells had increased farnesyl pyrophosphate synthase activity, although this was not due to upregulation of its gene transcription. Sensitivity differences to BPs may therefore result, at least in part, from increased activity of farnesyl pyrophosphate synthase [24].

It has been shown that overexpression or transformation of rodent or human cells by Ras results in cell lines that are substantially more resistant to radiation than are the parental cells [25]. Conversely, inhibition of Ras activation resulted in radiosensitization both in rodent cells transfected with Ras and in human tumor cells bearing endogenous mutations in Ras [26]. The radiosensitizing effect of BPs could therefore also be attributable to Ras signaling blockade by depleting the cellular pools of both geranylgeranyl pyrophosphate and farnesyl pyrophosphate.

Both prolonged G_2_M accumulation concomitant with an increase in susceptibility to induction of apoptosis and Ras signaling blockade may be associated with the cellular mechanisms of radiosensitization produced by BPs in tumor cells. In patients presenting with widespread bone metastases, retreatment of the same bone lesion is a factor that diminishes the quality of life. Adding zoledronic acid treatment to standard palliative radiotherapy might improve the latter's effectiveness. The outcome could be monitored in terms of mineralization, which represents healing of a particular metastasis. For breast carcinoma, combining standard radiation treatment with zoledronic acid might produce the same effect with a lower radiation dose, thus producing fewer side effects, lower fraction doses or lower total doses.

## Conclusion

The present study substantiates the synergistic cytotoxic effect of zoledronic acid combined with radiation on breast cancer cells. This *in vitro *finding is consistent with previous studies showing a synergistic effect of zoledronic acid with various chemotherapeutic agents. Turning to the clinical situation, the combination of zoledronic acid and radiotherapy might allow a reduction in radiation fraction doses or fraction numbers while maintaining the therapeutic effect in metastatic breast carcinoma patients.

## Abbreviations

BP = bisphosphonate; CI = combination index; DMSO = dimethyl sulfoxide; IC_50 _= 50% growth inhibition; IC_75 _= 75% growth inhibition; MTT = 3-(4,5-dimethyl-thiazoyl)-2,5-diphenyl-SH-tetrazolium bromide; PBS = phosphate-buffered saline.

## Competing interests

The authors declare that they have no competing interests.

## Authors' contributions

AUU designed the experiments, performed the analysis and interpretation of data and drafted the manuscript. FA carried out cell culture experiments and critically revised the manuscript. MC carried out cell culture experiments and performed cell growth determination. MG performed the radiation experiments. MAO participated in the CI calculation and revised the manuscript. All authors read and approved the final manuscript.
